# Synergistic Toxicity Interactions between Plant Essential Oil Components against the Common Bed Bug (*Cimex lectularius* L.)

**DOI:** 10.3390/insects11020133

**Published:** 2020-02-19

**Authors:** Sudip Gaire, Michael E. Scharf, Ameya D. Gondhalekar

**Affiliations:** Center for Urban and Industrial Pest Management, Department of Entomology, Purdue University, West Lafayette, IN 47907, USA; sgaire@purdue.edu (S.G.); mscharf@purdue.edu (M.E.S.)

**Keywords:** *Cimex lectularius* L., plant essential oils, monoterpenoids, bifenthrin, imidacloprid, synergism, electrophysiology

## Abstract

Management of the common bed bug (*Cimex lectularius* L.) necessitates the use of multiple control techniques. In addition to synthetic pesticides and mechanical interventions, plant-derived essential oils represent one of the control options. Mixtures of two or more essential oil components (monoterpenoids) exhibit synergistic toxicity effects against insects due to increased cuticular penetration. Monoterpenoids, such as carvacrol, eugenol and thymol, are neurologically active and inhibit the nerve firing activity of *C. lectularius*. However, the effects of mixtures of these monoterpenoids on their toxicity and neuroinhibitory potential against *C. lectularius* are not known. In this study, the toxicity levels of a tertiary mixture of carvacrol, eugenol and thymol (1:1:1 ratio) and a binary mixture of synthetic insecticides, bifenthrin and imidacloprid (1:1 ratio) were evaluated against *C. lectularius* through topical bioassays and electrophysiology experiments. Both a mixture of monoterpenoids and the mixture of synthetic insecticides exhibited synergistic effects in topical bioassays. In electrophysiology experiments, the monoterpenoid mixture led to greater neuroinhibitory effects, whereas a mixture of synthetic insecticides caused higher neuroexcitatory effects in comparison to single compounds. This study shows evidence for neurological mechanisms of synergistic interactions between monoterpenoids and provides information regarding the utilization of natural compound mixtures for *C. lectularius* management.

## 1. Introduction

The common bed bug (*Cimex lectularius* L.) is one of two *Cimex* spp. that has resurged globally in the last two decades as a pest of public health and economic importance [1]. Several hypotheses have been proposed to explain the resurgence of common bed bugs (hereafter referred to as bed bugs), including increased travel by the public and the evolution of pyrethroid insecticide resistance in field populations [2,3,4,5,6]. More recently, some bed bug populations were shown to be highly resistant to various neonicotinoids [7] and possess reduced susceptibility to pyrrole compounds (i.e., chlorfenapyr) [5]. Difficulty in eliminating resistant bed bug populations demands a multi-faceted pest management approach that utilizes both chemical and non-chemical or alternative treatment options [8,9,10]. Plant essential oils, which are secondary metabolites derived from internal and external glandular cells on the leaves and stems of aromatic plants [11], are one of the alternative treatment options used for the control of bed bugs, cockroaches and many other urban and agricultural pests [12,13,14,15,16,17,18,19]. More than 20 plant essential oils and their components are listed as minimum risk pesticides by the Environmental Protection Agency and are exempt from registration requirements (https://www.epa.gov/minimum-risk-pesticides; located in 40 CFR 152.25 (f)). Due to this exemption, many essential oil-based products are readily available in the market for the control of bed bugs and other urban pests. However, out of the nine essential oil products tested by Singh et al. [15] against bed bugs, only two were efficacious. This finding by Singh et al. [15] suggested that more in-depth research on the toxicology of essential oils is required to assist in the development of effective natural product formulations for bed bug and structural pest control in general.

Plant essential oils are composed of complex mixtures of monoterpenoids (generally referred to as essential oil components, compounds or constituents) with various functional groups, such as phenols, ketones, hydrocarbons, acids, etc. [11]. Of the various monoterpenoids tested in a recent study, the phenolic compounds carvacrol, eugenol and thymol were found to possess potent insecticidal activity against insecticide susceptible bed bugs when applied topically and/or as fumigants [20]. Furthermore, electrophysiology studies showed that these compounds also cause neuroinhibitory effects, i.e., suppression of the nervous system or nerve firing activity [20]. Additionally, target site studies conducted with carvacrol, eugenol and thymol suggested that they act on nicotinic acetylcholine (nACh), octapamine and gamma amino butyric acid (GABA) receptors, respectively [21,22,23].

Mixtures of two or more essential oil components exhibit synergistic, additive and/or antagonist toxicity effects in different insects, such as spider mites, cabbage loopers, house flies and nematodes [24,25,26,27]. Enhanced cuticular penetration caused by changes in pharmacokinetic properties (e.g., solubility and surface tension) of essential oil mixtures leads to synergistic action against the cabbage lopper [28,29]. While it is important to know all types of interactions between the monoterpenoids, synergistic toxicity interactions are more relevant from the perspective of pest management and the development of effective natural product formulations. Using synergistically interacting monoterpenoids in mixture products would allow us to achieve higher mortality by using smaller quantities of active ingredients [26,28]. In bed bugs, the synergistic, additive or antagonistic effects of essential oil component mixtures on the insect nervous system and at the bioassay level have not been determined, thereby representing a knowledge gap.

Given the neuroinhibitory effect of carvacrol, eugenol and thymol on bed bug ventral nerve cord activity (i.e., a ganglionic mass or fused thoracic and abdominal ganglia, as termed by Usinger [30]) [20] and their ability to act on different neuronal target sites [21,22,23], we hypothesized that an equal ratio mixture of these three compounds would cause additive or synergistic toxicity effects and lead to a greater neurophysiological impact against bed bugs. To test these hypotheses, the objectives of our study were to determine the impacts of an equal ratio mixture of carvacrol, eugenol and thymol on their (i) efficacy against bed bugs and (ii) neuroinhibitory effects on the bed bug nervous system. An equal ratio mixture of synthetic insecticides, bifenthrin (pyrethroid insecticide) and imidacloprid (neonicotinoid insecticide) was used as a positive control.

## 2. Materials and Methods

### 2.1. Insects

An insecticide-susceptible bed bug strain (Harlan) was used in this study. This strain was originally collected from the field in 1973 and has been maintained in the laboratory without insecticide selection pressure for more than 40 years. Insects were maintained in reach-in environmental chambers (Percival Scientific, Perry, IA, USA) at 25 °C temperature, 50% ± 15% relative humidity and a photoperiod of 12:12 (L: D) h. Insects were fed defibrinated rabbit blood (Hemostat Laboratories, Dixon, CA, USA) using the membrane feeding method described by Chin–Heady et al. [31]. Topical application bioassays were performed with 8–10 day old adult males (average weight = ~2 mg per insect) that were fed 4–5 days before initiating the bioassays. For neurophysiology experiments, 10–15 day old adult males were used. They were starved for 7–8 days before dissection. Starvation for longer durations decreased the amount of undigested blood in their gut and resulted in cleaner dissections [20].

### 2.2. Chemicals

The essential oil components carvacrol (≥98% purity) and eugenol (99% purity) were obtained from Sigma-Aldrich (St. Louis, MO, USA), whereas thymol (≥99% purity) was obtained from Alfa Aesar (Hill, MA, USA). Bifenthrin (98% purity) and imidacloprid (99.40% purity) were purchased from Chem Service Inc. (West Chester, PA, USA). Acetone, dimethyl sulfoxide (DMSO), buffer salts and other reagents used in neurophysiology experiments were purchased from either Sigma-Aldrich, Fisher Scientific (Hampton, NH, USA) or Avantor Performance Materials, LLC (Center Valley, PA, USA).

### 2.3. Topical Application Bioassays

The topical median lethal dose (LD_50_) values of the individual compounds carvacrol, thymol, eugenol and bifenthrin for the same bed bug strain (Harlan) were previously determined by Gaire et al. [20]. The LD_50_ estimates of imidacloprid, the tertiary mixture (1:1:1 ratio) of carvacrol, eugenol and thymol and the binary mixture (1:1 ratio) of bifenthrin and imidacloprid were determined in this study. Before preparing a tertiary mixture, carvacrol and eugenol were individually diluted in acetone on a volume-to-volume basis to prepare stock solutions based on the density of each component (carvacrol = 0.976 g/mL, eugenol = 1.067 g/mL). However, a stock solution of thymol was prepared on a weight-per-volume basis, since it was in crystal form. Stock solutions of imidacloprid and bifenthrin (positive control) were also prepared in acetone (weight to volume basis) and then mixed in a 1:1 ratio. The range of test concentrations (at least 5 concentrations) of single and mixed components or insecticides were determined through preliminary screening (concentration range, carvacrol + thymol + eugenol: 4.1–41.66; imidacloprid: 0.00025–003125; bifenthrin + imidacloprid: 0.0000775–0.000625 µg/mg body weight). For topical applications, insects were dorsally attached to the adhesive side of a colored label tape (Fisher Scientific) for immobilization. Insecticidal solutions (volume 0.5 µL) were applied topically on the ventral metathorax using a 25 µL syringe (Hamilton, Reno, NV, USA) attached to a PB-600-1 repeating dispenser (Hamilton). Control insect groups were treated with 0.5 µL of acetone. Treated and control insects were transferred into 35 × 10 mm Petri dishes (Greiner Bio-One, Frickenhausen, Germany) and placed in an environmental chamber. Mortality scoring of all treated insects was done at 24 h post-treatment. Insects that were lying on their backs and/or unable to move upon prodding were scored as dead. Mortality was also assessed 48 h post-treatment to ensure that insect recovery from intoxication symptoms did not occur. In total, three replicates were performed for each concentration in dose-response bioassays (n = 30; 10 adult males per replicate). Overall, 210–240 bed bugs were used for the determination of LD for each single compound or mixtures of the compounds.

### 2.4. Neurophysiology Equipment and Recording

Procedures followed for neurophysiology equipment setup, bed bug dissections and nervous system electrical activity recordings were adopted from an earlier study [20]. In brief, the neurophysiology equipment setup consisted of three electrodes, recording, reference and ground, which were connected to the model 4001 capacitance compensation head stage (Dagan Inc., Minneapolis, MN, USA). The head stage was further serially connected to noise eliminator, amplifier and digitizing computer software (i.e., Chart version 3.5.7, ADInstruments, Milford, MA, USA). The pulled glass capillary for the recording electrode was filled with HEPES (4-(2-Hydroxyethyl)piperazine-1-ethanesulfonic acid)-buffered physiological saline (pH 7.1) [20,32,33] and was placed in gentle contact with the fused ganglionic mass with the help of a micromanipulator (model MNJR, World Precision Instruments, Sarasota, FL, USA). The reference electrode was identical, but placed in contact with the carcass. A ground electrode was placed in the dissection dish outside the bed bug carcass.

**Figure 1:**
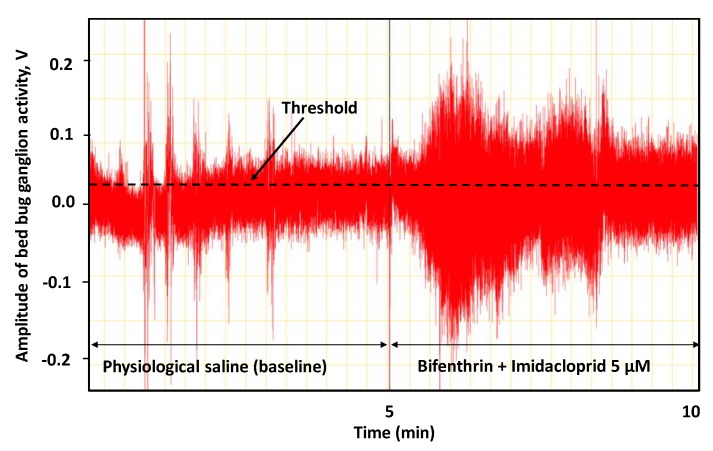
An example of a 10 min electrophysiological nerve activity recording for a 5 µM bifenthrin + imidacloprid mixture. Baseline spontaneous electrical activity recordings (pre-treatment) were performed in physiological saline for 5 min. After application of insecticide or mixture solutions to the nerve preparations, post-treatment recordings were performed for additional 5 min. The threshold was maintained at a constant level between the pre- and post-treatment recordings using the “counter” function in the Chart software.

Electrical activity recording with each insect was performed for 10 min (Figure 1). For the first 5 min, spontaneous pretreatment electrical activity (i.e., baseline) was recorded in physiological saline after setting a threshold level for the “counter” function on the Chart software (Figure 1). After 5 min, the recording was briefly paused to apply 1 µL of individual essential oil components (carvacrol, eugenol or thymol), diluted to 0.5 mM in physiological saline containing 0.1% DMSO and 0.01% Tween 20 or their tertiary mixture (1:1:1), gently onto the ganglion. For the mixture, each individual essential oil component solution (carvacrol, eugenol and thymol) was prepared at 3-fold higher concentration (1.5 mM); then, equal volumes of each component were mixed to obtain the final 0.5 mM mixture solution. The threshold for the “counter” function on the Chart software was maintained at a constant level for the 5 min pre- and 5 min post-treatment nerve activity recordings (Figure 1). For solvent control recordings, a solution containing physiological saline + 0.1% DMSO + 0.01% Tween 20 was used. The effect of solvent controls on nerve activity was compared to recordings that were conducted only in physiological saline during the 5-min pre- and post-treatment intervals. To determine the effect of individual compounds or their mixture on nerve activity, “departure ratios” were calculated by dividing the total number of spike counts surpassing the threshold in post-treatment recordings with the total number of spike counts above the threshold in pre-treatment or baseline recordings [20].

For the positive control treatments, bifenthrin, imidacloprid and their equal ratio mixture were tested at a concentration of 5 µM. However, the treatment volume was higher (2 µL) because the 1 µL volume was not effective [20]. Nine to ten replications or nerve preparations were performed for physiological saline, solvent control, each essential oil compound and their mixtures and all positive control treatments with synthetic insecticides. Each bed bug represented one replicate. If the bed bug died during the ten-minute recording period, that replicate was discarded and a new recording was performed with a new insect.

### 2.5. Statistical Analysis

The dose-mortality data for the essential oil constituent mixture, the positive control mixture, and imidacloprid were analyzed by probit analysis to calculate the LD_50_ values and their 95% fiducial limits (FL) [34]. Probit analysis was done using Minitab Software Release 14.2 (Minitab Inc., State College, PA, USA, released 2005). To determine the expected LD_50_ and interaction between essential oil compounds or synthetic insecticides in a mixture, we used Hewlett and Plackett’s model as per Tak et al. [26] and Tak and Isman [29].

$$E\;=\;(a\;\times \;{\mathrm{LD}}_{50}(a))\;+\;(b\;\times \;{\mathrm{LD}}_{50}(b))\;+\;(c\;\times \;{\mathrm{LD}}_{50}(c))\;+\ldots \ldots \ldots +(n\;\times \;{\mathrm{LD}}_{50}(n))$$

where E is Hewlett and Plackett’s expected LD_50_, a is the proportion of compound A in the mixture and LD_50_(a) is the LD_50_ of compound A, and so on. The interaction ratio was calculated by dividing the expected or theoretical LD_50_ value by the observed LD_50_. An interaction ratio greater than 1.5 indicates a synergistic interaction, a ratio of 1.5 or less and greater than 0.5 indicates an additive interaction and ratios of 0.5 or less indicate antagonism.

$$\mathrm{Interaction}\;\mathrm{ratio}\;(R)\;=\;\frac{\mathrm{Hewlett}\;\mathrm{and}\;\mathrm{Plackett}’s\;\mathrm{expected}\;\mathrm{LD}50\;\mathrm{of}\;\mathrm{mixture}}{\mathrm{Observed}\;\mathrm{LD}50\;\mathrm{of}\;\mathrm{mixture}}$$

For the neurophysiology data, departure ratios calculated for all mixtures or individual compounds were log-transformed after adding a value of one (1). The addition of the value “1” was done to obtain positive log-transformed values [20]. First, log-transformed departure ratios determined for the solvent controls were statistically compared to the physiological saline treatment using a two-sample t-test with Bonferroni’s adjusted significance level (0.05 divided by the number of comparisons or tests) [20,35,36]. Next, log-transformed departure ratio data for different mixtures and individual essential oil components or synthetic insecticides were compared to solvent controls using two-sample t-tests with Bonferroni’s adjusted significance level. Lastly, the same test was used to compare departure ratio data for single essential oil components or insecticides with their respective tertiary or binary mixtures. Two-sample t-tests were performed using SPSS (Statistical Product and Service Solutions) Version 25 (IBM Corp., Armonk, NY, USA, released 2017).

## 3. Results

### 3.1. Topical Toxicity

In all bioassays, <5% mortality was observed in acetone-treated bed bugs. The LD_50_ values of the individual compounds carvacrol, thymol, eugenol and bifenthrin, as determined by Gaire et al. [20], were 27.5, 32.5, 52 and 0.000345 µg/mg body weight, respectively. The LD_50_ value for imidacloprid was 0.0006 µg/mg body weight (Table 1). The tertiary mixture of carvacrol, thymol and eugenol caused a synergistic increase in bed bug mortality (interaction ratio of 1.96; Table 1). The mixture of bifenthrin and imidacloprid also showed synergism against bed bugs with an interaction ratio of 1.88 (Table 1).

### 3.2. Neurophysiological Effects of Mixtures

**Figure 2:**
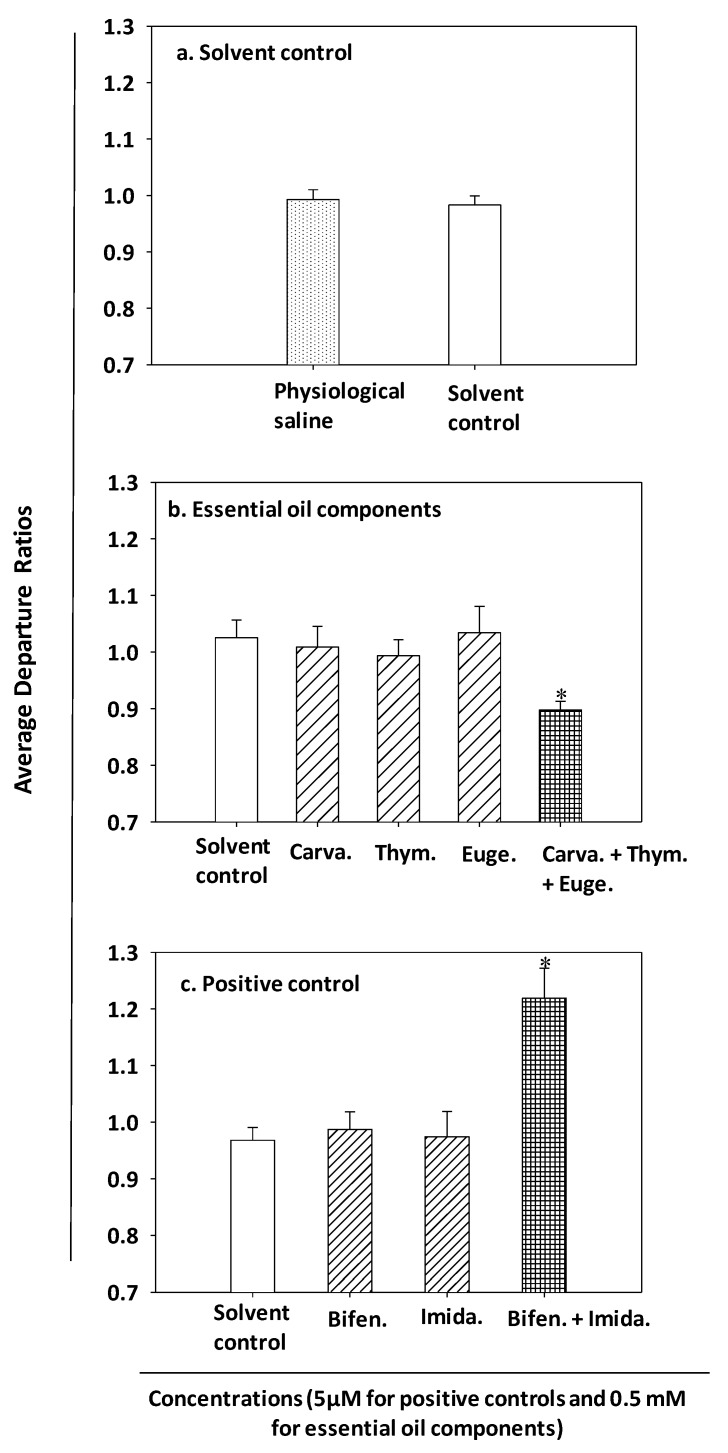
Neurophysiological effects of solvent control, essential oil constituents and positive control treatments on bed bug nervous system activity. Asterisks (*) in different graphs indicate significant differences compared to the solvent control recordings (two-sample t-tests with Bonferroni’ corrected *p*-value, i.e., 0.05 ÷ number of comparisons for each compound or mixture). (**a**) Solvent controls containing 0.1% dimethyl sulfoxide (DMSO) and 0.01% Tween 20 had no effect on nervous system activity (*p* > 0.05). (**b**) The essential oil component mixture of carvacrol (carva.), thymol (thym.) and eugenol (euge.) at 0.5 mM induced higher neuroinhibitory impacts than any of the individual compounds compared to the solvent control (*p* < 0.0125). (**c**) The positive control treatment mixture of bifenthrin (bifen.) and imidacloprid (imida.) at 5 µM induced significantly greater neuroexcitatory effects than either of the individual compounds in comparison to the solvent control (*p* < 0.016).

The solvent control treatment had no effect on bed bug nervous system activity in comparison to physiological saline (*p* = 0.682) (Figure 2a). When tested individually, the three essential oil components carvacrol, eugenol and thymol did not produce statistically significant inhibitory effects (i.e., no suppression of nerve firing activity) at the 0.5 mM concentration in comparison to the solvent control (carvacrol *p*-value = 0.435, thymol *p*-value = 0.468 and eugenol *p*-value = 0.918; Figure 2b). However, the mixture of the three essential oil components at the same 0.5 mM concentration inhibited spontaneous nerve firing by 12.44% when compared the solvent control (*p* = 0.003, two-sample *t*-test at Bonferroni’s corrected significance level of *p* < 0.0125) (Figure 2b). When neuroinhibitory effects of the tertiary mixture were compared to impacts caused by individual compounds, statistically significant differences were observed for all compounds (two-sample t-test at Bonferroni’s significance level of *p* < 0.016) (Figure 2b). More specifically, the neuroinhibitory potential of the tertiary mixture were 12%, 15% and 11% higher in comparison to the effects of the individual carvacrol, eugenol and thymol compounds, respectively (Figure 2b).

In the positive control treatment, the mixture of bifenthrin and imidacloprid at 5 µM produced significant neuroexcitation, i.e., a 25.94% increase in nerve firing activity compared to the solvent control treatment (*p* = 0.001, two-sample t-test at Bonferroni’s significance level of *p* < 0.016) (Figure 2c). However, when these insecticides were tested individually at 5 µM, they did not cause statistically significant overstimulation or neuroexcitation in comparison to the solvent control treatment (bifenthrin *p*-value = 0.669 and imidacloprid *p*-value = 0.967; Figure 2c). In contrast, the neuroexcitatory effect of the bifenthrin and imidacloprid mixture was significantly higher than the impacts of the individual insecticides (two-sample t-test at Bonferroni’s significance level of *p* < 0.025) (Figure 2c).

## 4. Discussion

Toxicity interactions between various compounds in insecticide mixtures are determined by a series of complex actions and counteractions between toxins and insect tissues [28]. Toxicity of insecticidal compounds or their mixtures is generally dependent upon cuticular penetration, activation of target sites and detoxification [28,37]. In this study, we observed that a tertiary mixture of carvacrol, eugenol and thymol led to a synergistic increase in their topical toxicity levels against bed bugs. These bioassay findings correlated with electrophysiology results, wherein the same tertiary mixture caused a significant decrease in nerve firing activity of fused thoracic and abdominal ganglia in comparison to the effects caused by the individual compounds (carvacrol, eugenol and thymol) at 0.5 mM concentration. In the following subsections, factors responsible for synergism between essential oil components at the sub-organismal (nervous system) and organismal (topical bioassays) levels are discussed, along with the implications of these findings for natural product development and bed bug management.

### 4.1. Mechanisms of Synergism between Monoterpenoids

Previous studies showed that a binary mixture of camphor and 1,8-cineole exhibited enhanced cuticular penetration, leading to a synergistic increase in toxicity against cabbage looper larvae [28]. These changes in the cuticular penetration ability of camphor and 1,8-cineole mixture are caused by pharmacokinetic factors that reduce surface tension and increase their solubility [28]. In addition to cuticular penetration-related mechanisms of synergism, the synergistic interaction that we observed between the tertiary mixture of carvacrol, eugenol and thymol was likely caused by target site-associated factors, such as the ability of the monoterpenoids to act on different target sites within the insect nervous system. As shown in Table 2, carvacrol, eugenol and thymol bind to nACh, octopamine and GABA receptors, respectively [21,22,23,38,39,40].

Carvacrol, eugenol and thymol also have similar effects on suppressing nerve firing activity of the bed bug nervous system at specific concentrations (Table 2) [20]. In general, neurologically active insecticides kill insects by inhibiting or overstimulating the normal firing activity of the nervous system [41,42,43,44,45]. Therefore, the simultaneous action of the tertiary mixture constituents at different binding sites is at least partially responsible for the suppression of nerve firing activity and the increased mortality observed in topical bioassays. Furthermore, changes in solubility, decreased surface tension and altered lipophilicity of essential oil constituent mixtures may allow them to penetrate the nervous system membrane more effectively, thus leading to greater neurophysiological effects.

Since essential oil components are volatile and exhibit vapor toxicity against various urban and agricultural insect pests [12,20,46], the effects of monoterpenoid mixtures on their vapor toxicity levels need to be determined in the future. Lastly, an increasing body of literature suggests that plant essential oils containing monoterpenoids inhibit cytochrome P450s in different mosquito species [47,48,49]. Thus, increased inhibition of detoxification enzymes by monoterpenoid mixtures could be yet another mechanism of synergism.

Topical bioassays and electrophysiology experiments that we conducted with *C. lectularius* using an equal ratio mixture of bifenthrin and imidacloprid (i.e., the positive control treatment), revealed a significant synergistic interaction between these two insecticides in whole organism bioassays and sub-organismal nerve activity recordings. Due to the differences in binding sites for pyrethroids (voltage-gated sodium channels) [44] and neonicotinoids (post-synaptic nAChRs) [42] and their neuroexcitatory actions (Table 2), it is expected that mixing insecticides from these two classes would cause a synergistic increase in activity toward target insect pests in comparison to either of the individual chemicals. Our findings regarding the synergism between pyrethroids and neonicotinoids were in agreement with the synergistic effects of a bifenthrin and imidacloprid mixture reported against mole crickets [43], wherein electrophysiology experiments showed that the mixture of bifenthrin and imidacloprid at a 10 µM concentration potentiated/synergized nerve firing activity of mole crickets and resulted in faster mortality in bioassays [43]. Neurological synergism between pyrethroids and other neuroexcitatory insecticides was also demonstrated in American cockroaches by Corbel et al. [50], who reported that a mixture of pyrethroid (permethrin) and carbamate (propoxur) insecticides drastically increased acetylcholine concentrations within the synaptic cleft.

### 4.2. Implications for Natural Product Development and Bed Bug Management

The use of synthetic organic insecticide mixtures is one of the strategies recommended for combating pesticide resistance in insect pests, including bed bugs [8,51]. Many laboratory and field-based studies with bed bugs showed that pyrethroid and neonicotinoid combination products exhibited higher efficacy against pyrethroid-resistant bed bugs and their eggs [52,53,54]. Several natural product insecticides containing a mixture of different essential oils (e.g., clove, cinnamon, cedar, peppermint, rosemary etc.) or their major constituents (e.g., eugenol and geraniol) are available in the market for bed bug control [15]. However, most of the available essential oil products are not effective against bed bugs [15], likely because they were formulated without considering synergistic, additive or antagonistic interactions that may occur either between different essential oils or their insecticidal components. The identification of the monoterpenoids carvacrol, eugenol and thymol, which interact synergistically and lead to increased toxicity against insecticide-susceptible bed bugs, is thus an important finding for informing the development of efficacious plant essential oil-based products for urban pest control. In the future, similar studies could be conducted with insecticide-resistant bed bug strains to determine the feasibility of using mixtures of different monoterpenoids for their control. Although there are limitations associated with the use of essential oils for urban pest control, such as odor and short residual activity, nanoformulated essential oils have less odor, are less volatile and show prolonged residual activity [55,56]. Additionally, in pesticide-susceptible and resistant American cockroaches and mosquitoes, essential oils were shown to potentially synergize the toxicity of pyrethroid and carbamate insecticides. either by inhibiting P450 enzymes or by activating neurological target sites [48,49,57]. Therefore, future research should also explore the possibility of using monoterpenoids or essential oils as synergists for overcoming resistance to pyrethroids and other insecticides in bed bugs.

## 5. Conclusions

The present study and previous research [20] collectively provide new insights into essential oil constituents that can be formulated together in botanical insecticide products. Furthermore, the identification of increased neuroinhibitory effects of a tertiary mixture of carvacrol, eugenol and thymol on the bed bug nervous system further advances our understanding of the mechanisms of synergistic interactions between monoterpenoids. Increased cuticular penetration [28], as well as a greater alteration of nerve firing activity than just additive, appear to be the major mechanisms responsible for synergism between monoterpenoids and essential oil components.

## Figures and Tables

**Table 1:** Topical toxicity of monoterpenoids, synthetic insecticides and their respective binary and tertiary mixtures against adult male bed bugs of the Harlan susceptible strain.

Treatments	N	Observed LD_50_^I^, µg/mg body weight (Fiducial Limits)	Expected LD_50_^II^, µg/mg body weight	Ratio (Interaction)
**Essential oil components**				
Carvacrol ^III^	−	27.5	−	−
Thymol ^III^	−	32.5	−	−
Eugenol ^III^	−	52	−	−
Carvacrol + thymol + eugenol	240	19 (17–21.5)	37.25	1.96 (Synergistic)
**Synthetic insecticides**				
Bifenthrin ^III^	−	0.000345	−	−
Imidacloprid	210	0.0006 (0.0005–0.00075)	−	−
Bifenthrin + imidacloprid	210	0.00025 (0.00025–0.0003)	0.00047	1.88 (Synergistic)

^I^ Observed median lethal dose (LD_50_) refers to the lethal dose required to kill 50% of the population, as calculated from the probit analysis. ^II^ Expected LD_50_ refers to the estimated LD_50_ values from Hewlett and Plackett’s model, as per Tak et al. [26] and Tak and Isman [29]. ^III^ LD_50_ values for carvacrol, eugenol, thymol and bifenthrin were adapted from Gaire et al. [20].

**Table 2:** Information regarding the target sites and neurological effects caused by plant essential oil components and synthetic insecticides used in the current study.

Essential Oil Components	Target Site	Neurological Effect	Effective Concentrations and Insect Species
Carvacrol	Nicotinic acetylcholine receptor (nAChR) [22]	Neuroinhibition [20]	4 mM in *Cimex lectularius* L. [20]
Thymol	Gamma-amino butyric acid receptor (GABA) [23]	Neuroinhibition [20]	4 mM in *C. lectularius* [20]
Eugenol	Octopamine receptor [21]	Neuroinhibition [20,41]	2 mM in *C. lectularius* [20]; 1 and 2 mM in *Periplaneta americana* L., *Blaberus discoidalis* Serville [41]
**Synthetic insecticides**			
Bifenthrin	Voltage-gated sodium channel [42]	Neuroexcitation [20,43]	10 µM in *C. lectularius* and *Scapteriscus vicinus* Scudder [20,43]
Imidacloprid	Nicotinic acetylcholine receptor [44]	Neuroexcitation [43]	10 µM in *S. vicinus* [43]
